# Identification of gaps in the current allergic rhinitis guidelines and how these can be filled

**DOI:** 10.1186/2045-7022-5-S4-P39

**Published:** 2015-06-26

**Authors:** Jean Bousquet, Claus Bachert, Ralph Mösges, Wytske Fokkens, Peter Hellings

**Affiliations:** 1 University Hospital of Montpellier, MACVIA-LR, EIP on Active & Health Ageing Ref Site, Montpellier, France; 2 University Hospital Ghent, Department of Oto-Rhinolaryngology, Ghent, Belgium; 3 University of Cologne, IMSIE, Cologne, Germany; 4 Academic Medical Center, Department of Otorhinolaryngology, Amsterdam, Netherlands; 5 University Hospitals Leuven, Dept of Otorhinolaryngology, Head & Neck Surgery, Leuven, Belgium

## 

Currently, there are gaps in the allergic rhinitis (AR) guidelines (and the evidence upon which they are based) that impede their function. Firstly, there is a scarcity of high quality evidence supporting AR treatment decisions [1]. Randomized controlled trials (RCTs) often fail to compare active treatments and use endpoints that are regulatory (not patient) driven. Secondly, a common AR control language for both patients and physicians and a common concept of AR control is missing. Finally, to date, the guidelines do not draw on evidence from real-life studies. These are important as they determine the effects of treatment under the usual conditions of care, thus maximizing applicability of findings to everyday practice. Many of these gaps are currently being filled. Firstly, the efficacy and safety of MP29-02* (a novel intranasal formulation of azelastine hydrochloride and fluticasone propionate in an advanced delivery system) has been shown versus active comparators in large, good quality, RCTs [2,3]. MP29-02*’s superiority over currently considered gold standard therapy (INS) was assessed using more clinically-relevant efficacy endpoints, defining treatment response in a way that is understandable and relevant to patients and health care providers, helping to explain why current first-line therapy often provides sub-optimal symptom relief. Secondly, a simple visual analog scale (VAS) has recently been proposed by MACVIA-LR ARIA as the control language of AR. This will form the basis for the new AR guidelines as part of an integrated care pathway (ICP) [4]. This VAS is also an integral component of a new app called Allergy Diary, designed to assess and track disease control. AR control has been categorized using VAS cut-off scores as ‘well-, partly- and un-controlled’. Finally, real-life studies are slowly gaining acceptance. One such study recently showed effective and rapid symptom control by MP29-02* in AR patients in real-life, using the same VAS score advocated in the ICP. Filling these gaps will ensure that treatment decisions are clinically relevant, encourage simplification of and compliance with AR management guidelines, ensure open and effective communication between all stakeholders and facilitate tailoring of AR medication to patients’ needs.

*Dymista

## References

[B1] Brozek JL Bousquet J Baena-Cagnani CE Bonini S Canonica GW Casale TB JACI 2010 126 3 466 476 10.1016/j.jaci.2010.06.047 20816182

[B2] Carr W Bernstein P Lieberman Meltzer E Bachert C Price D JACI 2012 129 5 1282 1289 10.1016/j.jaci.2012.01.077 22418065

[B3] Meltzer E Ratner P Bachert C Carr W Berger W Canonica GW Int Arch Allergy Immunol 2013 161 4 369 377 10.1159/000351404 23652808

[B4] Bousquet J Addis A Adcock I Agache I Agusti A Alonso A ERJ 2014 44 2 304 323 10.1183/09031936.00014614

